# Using pre-operative radiomics to predict microvascular invasion of hepatocellular carcinoma based on Gd-EOB-DTPA enhanced MRI

**DOI:** 10.1186/s12880-022-00855-w

**Published:** 2022-09-03

**Authors:** Xin-Yu Lu, Ji-Yun Zhang, Tao Zhang, Xue-Qin Zhang, Jian Lu, Xiao-Fen Miao, Wei-Bo Chen, Ji-Feng Jiang, Ding Ding, Sheng Du

**Affiliations:** 1 Department of Radiology, Nantong Third Hospital Affiliated to Nantong University, #60 Youth Middle Road, Chongchuan District, Nantong, Jiangsu China; 2 The First People’s Hospital of Taicang, Taicang, Suzhou, Jiangsu China; 3 Philips Healthcare, Shanghai, China

**Keywords:** Hepatocellular carcinoma, Microvascular invasion, Contrast media, Magnetic resonance imaging, Radiomics

## Abstract

**Objectives:**

We aimed to investigate the value of performing gadolinium-ethoxybenzyl-diethylenetriamine pentaacetic acid (Gd-EOB-DTPA) enhanced magnetic resonance imaging (MRI) radiomics for preoperative prediction of microvascular invasion (MVI) of hepatocellular carcinoma (HCC) based on multiple sequences.

**Methods:**

We randomly allocated 165 patients with HCC who underwent partial hepatectomy to training and validation sets. Stepwise regression and the least absolute shrinkage and selection operator algorithm were used to select significant variables. A clinicoradiological model, radiomics model, and combined model were constructed using multivariate logistic regression. The performance of the models was evaluated, and a nomogram risk-prediction model was built based on the combined model. A concordance index and calibration curve were used to evaluate the discrimination and calibration of the nomogram model.

**Results:**

The tumour margin, peritumoural hypointensity, and seven radiomics features were selected to build the combined model. The combined model outperformed the radiomics model and the clinicoradiological model and had the highest sensitivity (90.89%) in the validation set. The areas under the receiver operating characteristic curve were 0.826, 0.755, and 0.708 for the combined, radiomics, and clinicoradiological models, respectively. The nomogram model based on the combined model exhibited good discrimination (concordance index = 0.79) and calibration.

**Conclusions:**

The combined model based on radiomics features of Gd-EOB-DTPA enhanced MRI, tumour margin, and peritumoural hypointensity was valuable for predicting HCC microvascular invasion. The nomogram based on the combined model can intuitively show the probabilities of MVI.

**Supplementary Information:**

The online version contains supplementary material available at 10.1186/s12880-022-00855-w.

## Introduction

Hepatocellular carcinoma (HCC) is the most common primary cancer of the liver and accounts for approximately 75–85% of all liver cancers [1]. Although there are many effective treatment methods that can help control tumour progression and improve prognosis, such as surgery, liver transplantation, radiofrequency ablation, and transarterial chemoembolization, the recurrence rate of HCC remains high [2]. Microvascular invasion (MVI) is defined as a microscopic tumour thrombus in the vascular lumen lined by endothelial cells, and it occurs mainly in the branches of the peritumoural portal vein [3]. MVI is a recognised predictive factor for the survival of patients with HCC and recurrence after surgery [4–6]. Thus, the ability to preoperatively predict MVI would greatly benefit HCC treatment.

Radiologists can directly describe some semantic features to evaluate MVI, such as tumour size, non-smooth tumour margin, internal arteries, a hypodense halo, and tumour–liver differences [7–9]. However, evaluating these features is highly subjective, variable, and lacks robustness [10]. Radiomics has recently emerged and may be able to compensate for these shortcomings [11]. Radiomics can extract a large number of quantitative features from computed tomography (CT), magnetic resonance imaging (MRI), and ultrasound images that can be used to quantify the morphology or “imaging phenotype” of a tumour [12, 13]. The relationship between features found through tumour imaging and the underlying biology is indirect and complex [14]. Although the radiomics cannot quantify all relevant biological manifestations, the imaging phenotype can provide crucial supplementary information that can help guide treatment [15, 16].

A meta-analysis previously showed that MRI radiomics was better than CT radiomics for sensitivity, specificity, and the area under the receiver operating characteristic (ROC) curve (AUC) [17]. MRI can also provide better soft-tissue resolution, multi-parameters and more-stable features for assessing tumour heterogeneity [18–20]. The liver-specific contrast agent gadolinium-ethoxybenzyl-diethylenetriamine pentaacetic acid (Gd-EOB-DTPA) and the hepatobiliary phase (HBP) may provide additional information [21–25]. Therefore, this study aimed to build a model for predicting MVI by extracting and selecting crucial variables from multiple sequences of Gd-EOB-DTPA-enhanced MRI.

## Materials and methods

### Patient criteria

**Fig. 1:**
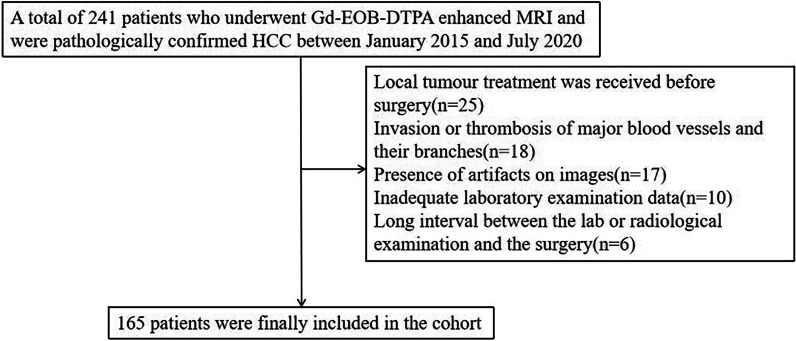
Flowchart of the enrollment of patients

We enrolled 165 patients who underwent partial hepatectomy for HCC between January 2015 and July 2020 in our hospital; these included 103 men and 62 women, aged 35–83 years, with a median age of 57 years. These patients met the following criteria (Fig. 1): (1) Gd-EOB-DTPA-enhanced MRI examination was performed within two weeks before surgery, (2) there was a postoperative pathological diagnosis of HCC, and (3) laboratory examination data were completed within two weeks before surgery. The exclusion criteria were: (1) local tumour treatment was received before surgery, including ablation therapy, radiotherapy, transarterial chemoembolization, and systemic therapy; (2) invasion or thrombosis of the portal vein, hepatic vein, inferior vena cava, and their main branches; (3) the presence of extrahepatic metastasis; and (4) the presence of artefacts on images. This retrospective study obtained ethical approval (approval number EK2021017), and the requirement for informed consent was waived.

### Clinical data

We collected data on age, sex, hepatitis B surface antigen, alpha-fetoprotein (AFP), alanine aminotransferase, aspartate aminotransferase (AST), albumin (ALB), total bilirubin, direct bilirubin, platelets, prothrombin time (PT), and international normalised ratio of each patient. Two experienced pathologists evaluated cirrhosis of the liver parenchyma and MVI according to the Standardization for Diagnosis and Treatment of Primary Hepatic Carcinoma in China (2019 edition). We divided all patients into an MVI-positive or MVI-negative group.

### MRI protocols

A Philips 3.0T Achieva MR scanner with a 16-channel abdominal coil was used to perform the abdominal MRI. The MRI plain scan was performed first, including T2-weighted imaging (T2WI) with spectral attenuated inversion recovery, diffusion-weighted imaging (DWI) (b = 0, 800 s/mm^2^), and in-phase/out-phase T1-weighted imaging (T1WI). The contrast agent Gd-EOB-DTPA (Germany Bayer, Medical Health Co., Ltd.) was injected into the cubital vein at a flow rate of 1.0 ml/s, using 0.1 ml/kg of bodyweight, and rinsed with 20 ml saline after injection. The enhancement scan used the T1 high-resolution isotropic volume excitation sequence (THRIVE). Axial images of the arterial phase (AP), portal venous phase (PP), transitional phase (TP), and HBP were collected at 20 s, 1 min, 3 min, and 20 min after injection of the contrast agent. The parameters of the scan sequences are listed in Table 1.

**Table 1:** The parameters of the scan sequences

	TR (ms)	TE (ms)	Slice thickness (mm)	Slice gap (mm)	Matrix
T2WI	2000	70.0	5.0	1.0	250 × 230
DWI	3000–5000	55.0	5.0	1.0	128 × 160
In-phase/out-phase T1WI	150	2.3/1.15	7.0	1.0	250 × 230
THRIVE	3	1.5	2.5	0	250 × 230

*T2WI* T2-weighted imaging, *DWI* diffusion-weighted imaging, *T1WI* T1-weighted imaging, *THRIVE* high-resolution isotropic volume excitation sequence

#### Image analysis

Two experienced radiologists, with more than eight years of experience in abdominal disease diagnosis and blinded to the pathology results, evaluated the radiological features and reached a consensus. We recorded the multifocality of the lesions, tumour size, signal uniformity on T2WI, peritumoural enhancement in the AP, tumour capsule in the PP, tumour margin, and peritumoural hypointensity in the HBP. A smooth tumour margin presents as a nodular tumour with smooth contour, while a non-smooth tumour margin is characterized by a lobulated tumour or irregular protrusions into the surrounding normal liver parenchyma. Peritumoural hypointensity is defined as an irregular, wedge-shaped, flame-like hypointense area of the liver parenchyma located outside of the tumour margin. The signal of the hypointense area is lower than that of the normal liver parenchyma but higher than that of the tumour itself. A few HCC lesions in the HBP show isointensity or hyperintensity; the signal intensity of the peritumoural hypointense area is lower than that of the lesion, but were less hypointense than the hypointense rim that is usually depicted in those HCCs [26]. When there were multiple lesions, the largest lesion was selected. A physician who was not involved in the radiological and radiomics analyses checked the radiological and pathological findings and marked the largest HCC lesion. These subjective radiological features and clinical data are collectively referred to as the clinicoradiological data.

### Drawing the regions of interest

**Fig. 2:**
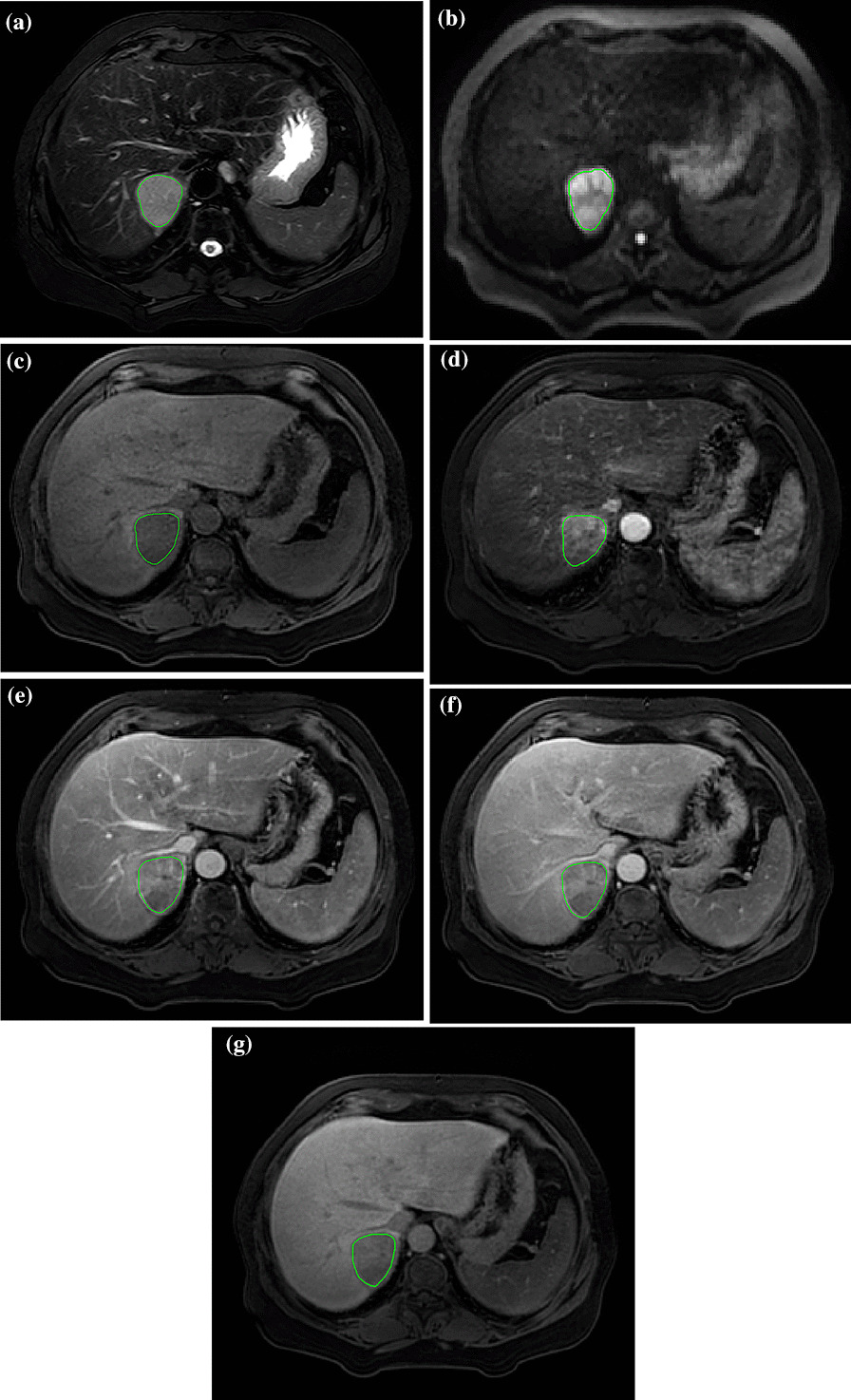
The delineation of ROI on T2WI (**a**), DWI (**b**), T1WI (**c**), AP (**d**), PP (**e**), TP (**f**) and HBP (**g**). The ROI was drawn as close to the margin of the tumour as possible but within the margin on the largest section

The original images were imported into the Philips Radiomics Tool 1.9.2 (Philips Investment Co., Ltd., Shanghai, China) software (based on Pyradiomics 3.0.0). The grey level normalization was performed before feature extraction, and the grey level discretization was undertaken using a bin width of 2.5. The first radiologist who was not involved in the radiological analysis manually delineated the region of interest (ROI) by selecting the largest tumour section on the T2WI, DWI (b = 800 s/mm^2^), T1WI plain scan, AP, PP, TP, and HBP images (Fig. 2). When multiple lesions were present, only the largest lesion was delineated. Another senior physician confirmed the ROI outlined by the first radiologist. Two months later, the first radiologist drew the ROI on images from 20 randomly selected patients, and these data were used to calculate the intraclass correlation coefficients (ICCs).

### Feature extraction

Feature extraction was performed using Philips Radiomics Tool software (based on Pyradiomics 3.0.0). Each sequence had 1227 features, included 17 shape features presented by statistical values, 19 first-order features that indicated the distribution of intensities, 75 texture features (included the grey-level size-zone matrix [GLSZM], grey-level run-length matrix [GLRLM], grey-level co-occurrence matrix [GLCM], neighbouring grey-tone difference matrix [NGTDM], and grey-level dependence matrix [GLDM]) that quantified the intratumoural heterogeneity, and 1116 first-order features and texture features that were obtained using exponential, logarithmic, squared, square root, or wavelet filtering.

### Feature selection

ICCs were used to evaluate the stability of each radiomics feature value. Features with an ICC ≥ 0.75 were used for further feature selection. The Z-score was used to standardise the features before putting them in the model. The overall cohort was divided at a ratio of 7:3 into a training set (n = 115) and a validation set (n = 50), and there were no significant differences in the baseline data between the training and validation sets. In the training set, stepwise regression was used to select the clinicoradiological variables. The least absolute shrinkage and selection operator (LASSO) algorithm was used to select important radiomics features by tenfold cross-validation. The LASSO algorithm was also used to determine variables from the combined dataset composed of the clinicoradiological data and radiomics features with ICC ≥ 0.75. The validation set was used to test the accuracy of the model predictions. A correlation coefficient heatmap was generated to show the correlation between clinicoradiological variables and radiomics features.

### Model construction and evaluation

Based on the selected variables, multivariate logistic regression was used to construct the clinicoradiological model, radiomics model, and combined model. The AUC, sensitivity, and specificity were used to evaluate the diagnostic performance of the models. Univariate logistic regression analysis was performed between the selected variables and the MVI. A nomogram risk-prediction model was constructed based on the variables included in the combined model. The nomogram model intuitively shows scores based on the coefficients of the logistic regression model variables and converts them into probabilities of clinical events. The nomogram model was internally validated using 1000 random bootstrap resamplings. The discrimination and calibration of the nomogram model was evaluated using the C-index and calibration curve.

### Statistical analysis

All statistical analyses were performed using the R software (version 3.6.0). The Kolmogorov–Smirnov test and analysis of variance were used to test for distribution normality and homogeneity of variance. The t-test or Mann–Whitney rank test was used to compare the differences in the quantitative data, and the chi-square test or Fisher’s exact test was used to compare the differences in the qualitative data. The LASSO algorithm and ROC curve were performed using the “glmnet” and “pROC” packages, and the “rms” package generated the nomogram and calibration curve. The correlation coefficient heatmap was drawn using the “corrplot” package. A *P* value < 0.05 indicated statistical significance.

## Results

### Patients

Among the 165 HCC patients, 49 were MVI positive and 116 were MVI negative. In the clinicoradiological model, radiomics model, and combined model, the baseline data between the training and validation sets were not statistically different (*P* > 0.05). The baseline data of the clinicoradiological model are shown in Table 2.

**Table 2:** Comparison of the baseline data of the clinicoradiological data

	Training set (n = 115)			Validation set (n = 50)			P*
	MVI-negative (n = 81)	MVI-positive (n = 34)	*P* value	MVI-negative (n = 35)	MVI-positive (n = 15)	*P* value	
Sex			1.00			0.30	0.65
0, Female	32 (39.5)	13 (38.2)		14 (40.0)	3 (20.0)		
1, Male	49 (60.5)	21 (61.8)		21 (60.0)	12 (80.0)		
Multifocality			1.00			1.00	0.55
0, No	69 (85.2)	29 (85.3)		28 (80.0)	12 (80.0)		
1, Yes	12 (14.8)	5 (14.7)		7 (20.0)	3 (20.0)		
Cirrhosis							
0, No	5 (6.2)	3 (8.8)	0.92	2 (5.7)	2 (13.3)	0.73	0.47
1, Yes	76 (93.8)	31 (91.2)		33 (94.3)	13 (86.7)		
HBsAg			0.64			0.93	0.96
0, Negative	14 (17.3)	4 (11.8)		5 (14.3)	3 (20.0)		
1, Positive	67 (82.7)	30 (88.2)		30 (85.7)	12 (80.0)		
Tumour signal on T2WI			0.06			1.00	0.67
0, Homogeneous	15 (18.5)	1 (2.9)		4 (11.4)	1 (6.7)		
1, Heterogeneous	66 (81.5)	33 (97.1)		31 (88.6)	14 (93.3)		
Peritumoural enhancement			0.65			0.05	0.45
0, No	71 (87.7)	28 (82.4)		31 (88.6)	9 (60.0)		
1, Yes	10 (12.3)	6 (17.6)		4 (11.4)	6 (40.0)		
Tumour capsule			< 0.001			0.34	0.17
0, Absent	25 (30.9)	8 (23.5)		11 (31.4)	3 (20.0)		
1, Incomplete	7 ( 8.6)	15 (44.1)		9 (25.7)	7 (46.7)		
2, Complete	49 (60.5)	11 (32.4)		15 (42.9)	5 (33.3)		
Tumour margin			< 0.001			0.10	0.07
0, Smooth	62 (76.5)	12 (35.3)		20 (57.1)	4 (26.7)		
1, Non-smooth	19 (23.5)	22 (64.7)		15 (42.9)	11 (73.3)		
Peritumoural hypointensity			< 0.001			0.01	0.51
0, No	75 (92.6)	17 (50.0)		30 (85.7)	7 (46.7)		
1, Yes	6 (7.4)	17 (50.0)		5 (14.3)	8 (53.3)		
Age	58.00 [52.00, 65.00]	60.00 [51.50, 64.00]	0.95	57.00 [50.00, 63.50]	55.00 [52.50, 63.50]	0.76	0.67
Tumour size	2.18 [1.60, 3.18]	3.68 [2.70, 5.79]	< 0.001	2.68 [1.95, 3.65]	4.20 [3.30, 5.33]	0.01	0.90
AFP	8.19 [3.32, 89.75]	25.98 [3.50, 183.14]	0.24	50.29 [6.24, 225.46]	22.14 [6.17, 82.26]	0.62	0.52
ALT	30.00 [22.00, 46.00]	28.50 [22.25, 46.00]	0.62	30.00 [23.50, 44.00]	30.00 [17.50, 46.50]	0.66	0.78
AST	36.00 [27.00, 50.00]	35.50 [26.50, 45.00]	0.58	38.00 [27.00, 53.50]	32.00 [26.00, 38.00]	0.22	0.88
ALB	42.10 [38.80, 44.30]	42.05 [38.25, 44.85]	0.69	40.60 [37.40, 44.55]	42.50 [39.75, 44.70]	0.44	0.81
TBIL	15.90 [12.70, 21.30]	15.40 [11.88, 18.78]	0.27	15.00 [11.95, 22.75]	16.50 [12.30, 20.95]	0.90	0.68
DBIL	5.30 [4.10, 7.10]	4.55 [4.03, 6.68]	0.26	5.40 [3.85, 7.90]	6.30 [3.40, 7.30]	0.97	0.43
PLT	111.00 [69.00, 144.00]	109.50 [82.75, 146.75]	0.32	107.00 [80.50, 127.00]	145.00 [102.50, 207.00]	0.04	0.34
PT	12.50 [11.60, 13.40]	11.80 [11.20, 12.33]	0.04	12.60 [11.90, 13.40]	11.90 [11.65, 12.40]	0.11	0.13
INR	1.07 [0.99, 1.17]	1.01 [0.96, 1.05]	0.02	1.08 [1.02, 1.15]	1.05 [1.00, 1.08]	0.15	0.45

P*, *P* value for the test between the training set and the validation set

*MVI* microvascular invasion, *HBsAg* hepatitis B surface antigen, *T2WI* T2-weighted imaging, *AFP* alpha-fetoprotein, *ALT* alanine-aminotransferase, *AST* aspartate-aminotransferase, *ALB* albumin, *TBIL* total bilirubin, *DBIL* direct bilirubin, *PLT* platelets, *PT* prothrombin time, *INR* international normalised ratio

### Construction of the clinicoradiological model

The tumour capsule, tumour margin, peritumoural hypointensity, tumour size, AST, ALB, and PT were selected as significant variables related to MVI. Multivariate logistic regression was used to construct the clinicoradiological model.

The formula of the model was as follows:

$$\begin{aligned} \log ({\text{p}}/(1 - {\text{p}})) & = 5.6642 - 0.6055 \times {\text{tumour capsule}} + {1}.{4956} \times {\text{tumour margin}} + {1}.{8169} \\ & \quad \times {\text{peritumoural hypointensity}} + 0.{2511} \times {\text{tumour size}} \\ & \quad - 0.0{233} \times {\text{AST}} - 0.0{998} \times {\text{ALB}} + {2}.0{827} \times {\text{PT}} \\ \end{aligned}$$

where *p* is the probability of MVI. The univariate logistic regression analysis of the variables in the validation set showed that peritumoural hypointensity had the highest odds ratio (OR) (Table 3).

**Table 3:** The univariate logistic regression analysis of each variable with OR in the validation set of the clinicoradiological model

	OR	95% CI	*P* value
Tumour capsule	0.55	0.30–0.98	0.006
Tumour margin	4.46	1.49–13.35	0.006
Peritumoural hypointensity	6.15	1.64–23.06	0.006
Tumour size	1.29	0.96–1.73	0.082
AST	0.98	0.94–1.01	0.143
ALB	0.90	0.79–1.04	0.095
PT	0.74	0.53–1.01	0.055

*OR* odds ratio, *CI* confidence interval, *AST* aspartate-aminotransferase, *ALB* albumin, *PT* prothrombin time

### Construction of the radiomics model

The proportion of radiomics features with an intra-observer ICC ≥ 0.75, 0.5–0.75, and < 0.5 were 86.35%, 10.48%, and 3.17%, respectively. The LASSO algorithm was used to select features with ICC ≥ 0.75 further. Ultimately, eight radiomics features were selected to construct the radiomics model; four were from the DWI, and the other four were from T2WI, PP, TP, and T1WI.

The formula of the model was as follows:

$$\begin{aligned} \log ({\text{p}}/(1 - {\text{p}})) & = - {8}.{6134} - \, 0.{3}0{37} \times {\text{TP}}\_{\text{Wavelet}}.{\text{GLDM}}.{\text{LLH}}\_{\text{DependenceVariance }}({\text{DV}}) \, \\ & \quad + {6}.{7528} \times {\text{PP}}\_{\text{GLCM}}\_{\text{InverseDifferenceNormalized }}({\text{IDN}}) \\ & \quad + 0.0{29}0 \times {\text{T1WI}}\_{\text{Exponential}}.{\text{GLDM}}\_{\text{DV }} \\ & \quad + 0.000{8} \times {\text{DWI}}\_{\text{Wavelet}}.{\text{FirstOrder}}.{\text{LLL}}\_{\text{Range}} \\ & \quad - \, 0.000{9} \times {\text{DWI}}\_{\text{Wavelet}}.{\text{GLSZM}}.{\text{HLL}}\_{\text{SizeZoneNon}} - {\text{uniformity }}({\text{SZN}}) \, \\ & \quad + 0.{138}0 \times {\text{DWI}}\_{\text{SquareRoot}}.{\text{GLSZM}}\_{\text{SZN }} \\ & \quad + 0.{3}0{4}0 \times {\text{DWI}}\_{\text{Logarithm}}.{\text{GLSZM}}\_{\text{SmallAreaHighGrayLevelEmphasis }}({\text{SAHGLE}}) \\ & \quad + 0.00{4}0 \times {\text{T2WI}}\_{\text{Wavelet}}.{\text{FirstOrder}}.{\text{HLL}}\_{\text{Maximum}} \\ \end{aligned}$$

The univariate logistic regression of the variables in the validation set showed that SquareRoot.GLSZM_SZN from the DWI had the highest OR, followed by Wavelet.FirstOrder.HLL_Maximum from the T2WI and Wavelet.GLSZM.HLL_SZN from the DWI (Table 4).

**Table 4:** The univariate logistic regression analysis of each variable with OR in the validation set of the radiomics model

Sequence	Feature	OR	95% CI	*P* value
TP	Wavelet.GLDM.LLH_DV	1.39	(1.01–1.91)	0.041
PP	GLCM_IDN	1.54	(1.11–2.14)	0.008
T1WI	Exponential.GLDM_DV	1.19	(1.01–1.40)	0.028
DWI	Wavelet.FirstOrder.LLL_Range	1.09	(0.46–2.58)	0.851
DWI	Wavelet.GLSZM.HLL_SZN	2.25	(0.83–6.07)	0.107
DWI	SquareRoot.GLSZM_SZN	9.12	(1.82–15.78)	0.004
DWI	Logarithm.GLSZM_SAHGLE	1.04	(1.02–1.06)	0.030
T2WI	Wavelet.FirstOrder.HLL_Maximum	2.70	(1.16–6.28)	0.012

DV measures the variance in dependence size in the image; ID normalizes the difference between the neighboring intensity values; Range represents the range of gray values in the ROI; SZN measures the variability of size zone volumes in the image; SAHGLE measures the proportion in the image of the joint distribution of smaller size zones with higher gray-level values; Maximum is the maximum gray level intensity within the ROI

*OR* odds ratio, *CI* confidence interval, *TP* transitional phase, *PP* portal venous phase, *T1WI* T1-weighted imaging, DWI,diffusion-weighted imaging, *T2WI* T2-weighted imaging, *GLDM* grey-level dependence matrix, *GLCM* grey-level co-occurrence matrix, *GLSZM* grey-level size-zone matrix, *DV* dependence variance, *IDN* inverse difference normalized, *SZN* size zone non-uniformity, *SAHGLE* small area high gray level emphasis

**Fig. 3:**
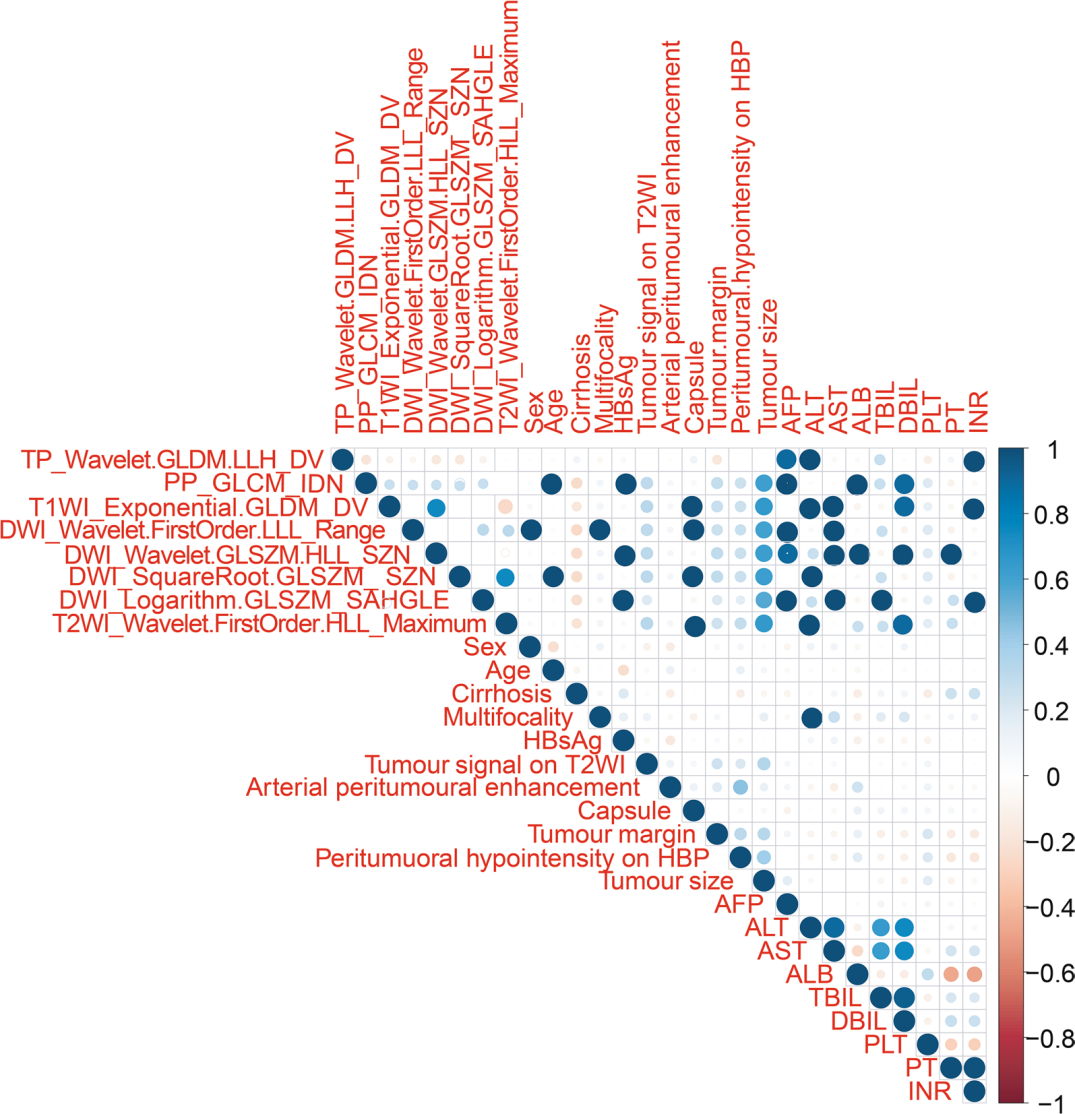
The correlation coefficient heatmap for clinicoradiological variables and radiomics features selected in the radiomics model. The larger the value or the darker the color is, the stronger the correlation is

The correlation between clinicoradiological variables and the above 8 radiomics features is shown in the correlation coefficient heatmap (Fig. 3).

### Construction of the combined model

**Fig. 4:**
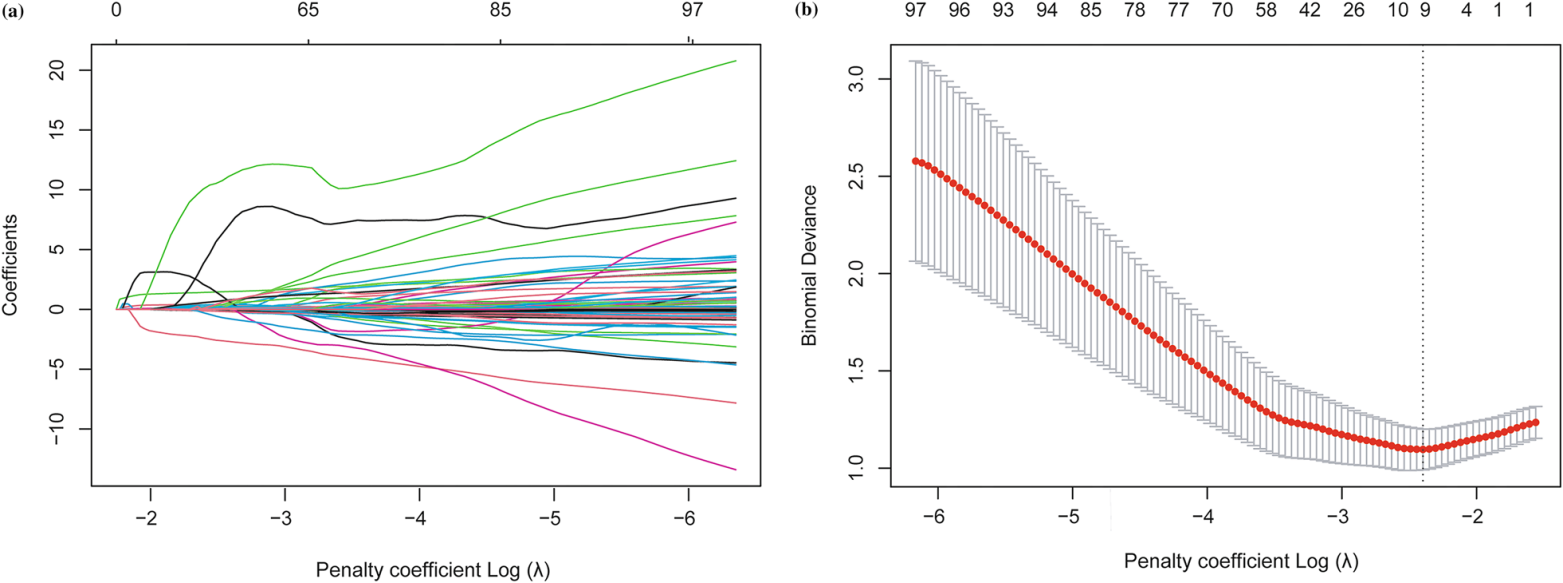
LASSO algorithm used for the combined model. **a** The variation of the coefficients of the variables with the penalty coefficient (λ) **b** Use tenfold cross-validation to select λ. When the binomial deviation was the smallest (minimum standard), nine nonzero coefficients were determined

The combined data comprised 20 clinicoradiological variables and radiomics features with ICC ≥ 0.75; the tumour margin, peritumoural hypointensity, and seven radiomics features were selected by the LASSO algorithm to construct the combined model (Fig. 4). The tumour margin and peritumoural hypointensity were significant variables in both the clinicoradiological model and the combined model. The size zone non-uniformity (SZN) from the DWI, small area high grey-level emphasis (SAHGLE), and Maximum from T2WI were significant variables in both the radiomics model and the combined model.

The specific formula of the model was:

$$\begin{aligned} \log ({\text{p}}/(1 - {\text{p}})) & = - {8}.{8963} + 0.{793}0 \times {\text{tumourmargin}} + {2}.{17}00 \times {\text{peritumoural hypointensity}} \\ & \quad - {11}.{3837} \times {\text{HBP}}\_{\text{logarithm}}.{\text{GLSZM}}\_{\text{SmallAreaLowGrayLevelEmphasis }}({\text{SALGLE}}) \\ & \quad - { 9}.{3923} \times {\text{PP}}\_{\text{wavelet}}.{\text{GLCM}}.{\text{HLL}}\_{\text{IDN }} \\ & \quad + {17}.{1969} \times {\text{DWI}}\_{\text{wavelet}}.{\text{FirstOrder}}.{\text{HLL}}\_{\text{Skewness }} \\ & \quad + 0.0{4}00 \times {\text{HBP}}\_{\text{shapebased}}\_{\text{MeshVolume }} \\ & \quad + 0.0{8}0{4} \times {\text{DWI}}\_{\text{squareRoot}}.{\text{GLSZM}}\_{\text{SZN }} \\ & \quad + 0.{2524} \times {\text{DWI}}\_{\text{logarithm}}.{\text{GLSZM}}\_{\text{SAHGLE }} \\ & \quad + 0.00{17} \times {\text{T2WI}}\_{\text{wavelet}}.{\text{Firstorder}}.{\text{HLL}}\_{\text{Maximum}} \\ \end{aligned}$$

**Fig. 5:**
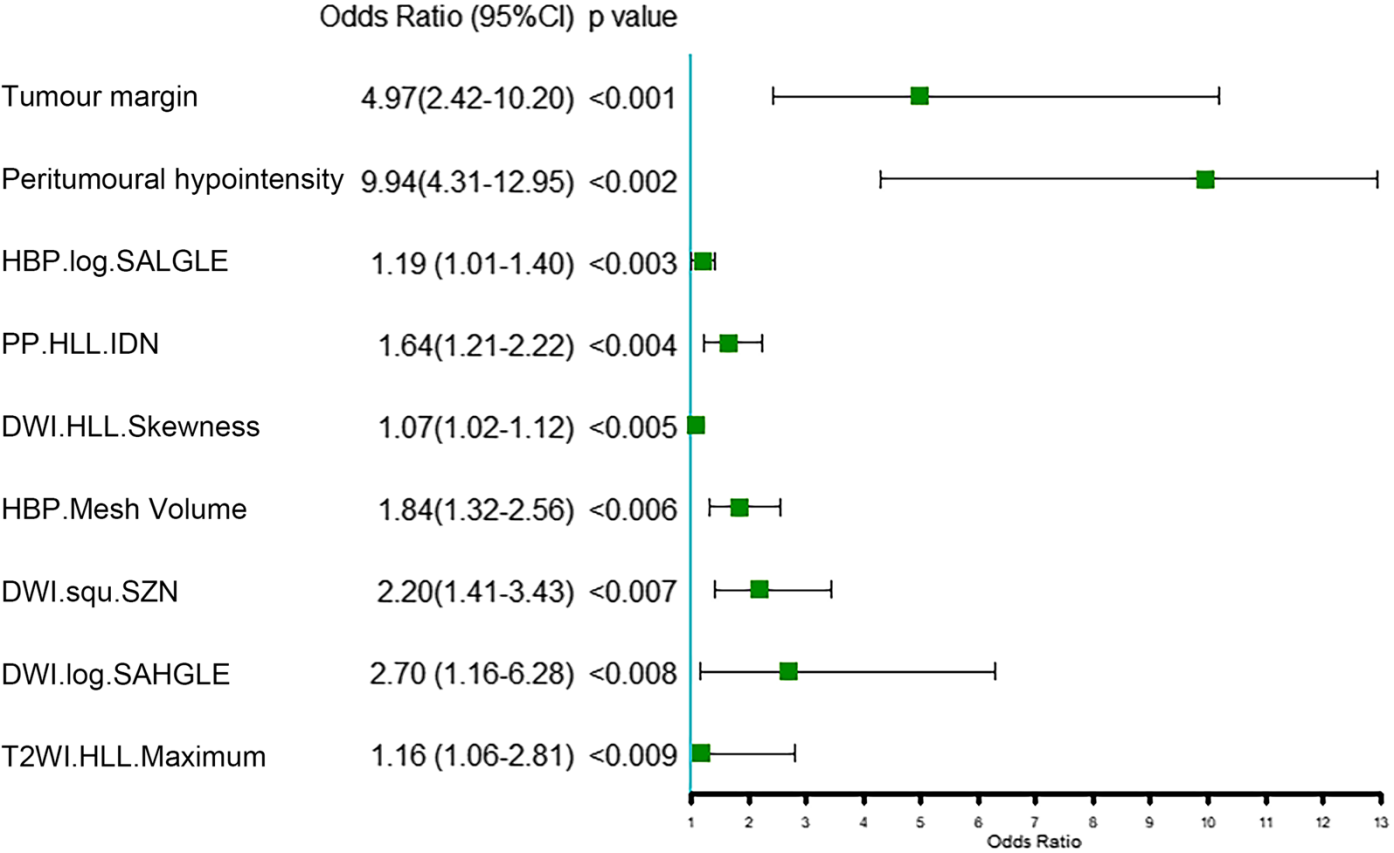
Forest plot showing the univariate logistic regression analysis of each variable with OR in the validation set of the combined model

The results of the univariate logistic regression in the validation set are presented in a forest plot in Fig. 5. Peritumoural hypointensity had the highest OR, followed by the tumour margin.

### Construction of the nomogram model

**Fig. 6:**
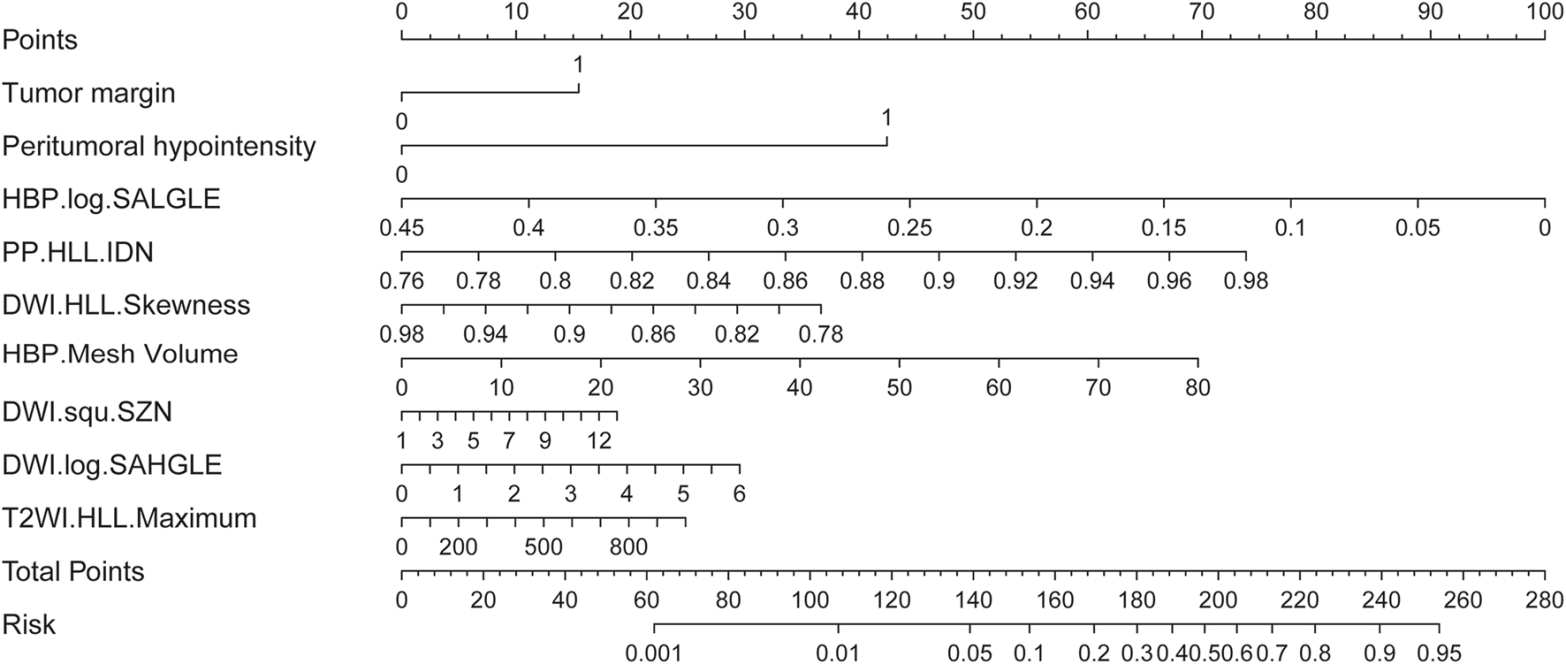
Construction of the nomogram. The point of each variable was added up to obtain the total points. The total point corresponded to the risk probability of predicting MVI. The nomogram can be used to predict the risk probability of MVI for each patient

**Fig. 7:**
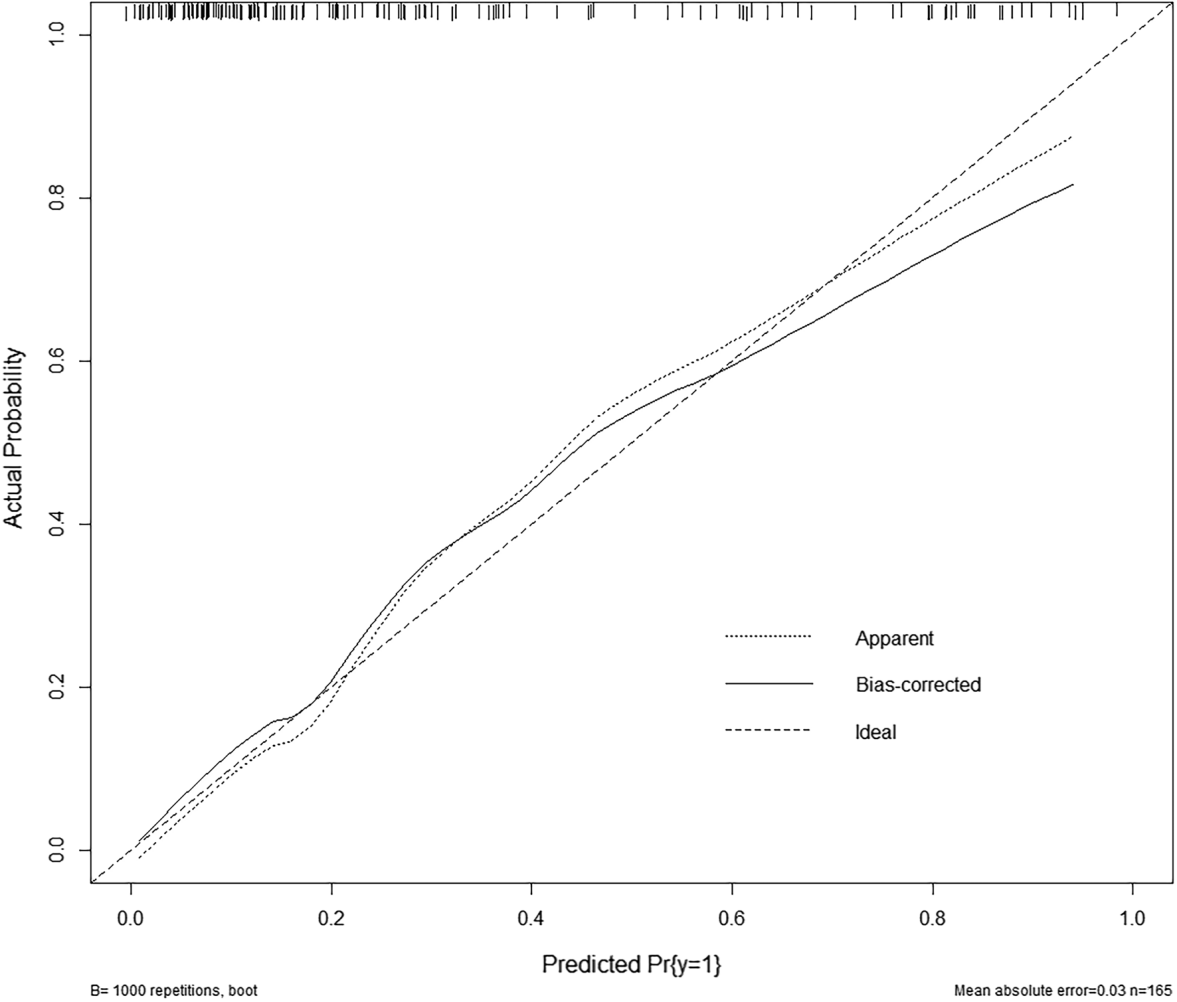
Calibration curve of the nomogram model

A nomogram risk-prediction model was established based on the variables in the combined model (Fig. 6). The C-index of the nomogram model was 0.79 (95% CI: 0.68–0.83). The calibration curve showed that the predicted probability from the nomogram model was close to the actual probability (Fig. 7). The mean absolute error between the predicted probability and the actual occurrence of MVI was 0.03, which indicated that the nomogram model had good calibration.

### Performance of the models

The performances of the three models for predicting MVI are shown in Table 5. The AUC of the combined model was higher than that of the clinicoradiological and radiomics models in the training set (0.841, 0.782, and 0.715, respectively). Similar results were observed in the validation set, with AUC values of 0.826, 0.755, and 0.708, respectively. The AUC of the radiomics model was higher than that of the clinicoradiological model in both the training and validation sets. The combined model had the highest sensitivity for predicting MVI (81.43% in the training set and 90.89% in the validation set); the clinicoradiological model had the highest specificity (91.67% in the training set and 92.07% in the validation set).

**Table 5:** The performances of the models in predicting MVI

	Training set					Validation set				
	AUC	95% CI	Sen (%)	Spe (%)	*P* value	AUC	95% CI	Sen (%)	Spe (%)	*P* value
Clinicoradiological model	0.715	0.678–0.745	51.88	91.67	0.003	0.708	0.677–0.719	50.33	92.07	0.041
Radiomics model	0.782	0.734–0.801	63.71	82.80	0.012	0.755	0.721–0.786	84.50	80.78	0.012
Combined model	0.841	0.822–0.867	81.43	72.76	0.007	0.826	0.798–0.838	90.89	69.85	0.026

*AUC* area under the ROC curve, *CI* confidence interval, *Sen* sensitivity, *Spe* specificity

## Discussion

In previous studies, the radiomics features from T2WI, T1WI, DWI, apparent diffusion coefficient (ADC) map, AP, and PP had specific values for predicting MVI [27–30]. However, there are few radiomics studies based on Gd-EOB-DTPA-enhanced MRI [21–25]. Although some studies selected features from the HBP and achieved excellent performance [21–23], models composed of features from multiple sequences of Gd-EOB-DTPA-enhanced MRI lacked discusssion. This study selected radiomics features from multiple sequences and used logistic regression to establish models for predicting MVI in HCC. Contrary to previous studies [21–23], the combined model of this study used the LASSO algorithm to select features from the combined dataset to avoid the collinearity between the clinicoradiological variables and radiomics features. We also used a correlation coefficient heatmap to show the possible correlation between the clinicoradiological variables and radiomics features.

The results showed that, among the variables included in the clinicoradiological model, only the tumour margin and peritumoural hypointensity in the HBP were stable in the combined model. In the radiomics model, the SZN from DWI, SAHGLE, and Maximum from T2WI were important radiomics features in the combined model.

In both the clinicoradiological model and the combined model, the OR for the tumour margin and peritumoural hypointensity were significantly higher than that of the other variables in the validation set. Several studies have demonstrated that peritumoural hypointensity in the HBP is an independent risk factor for predicting MVI [26, 31]. Peritumoural hypointensity may be related to peritumoural hemodynamic changes caused by the tumour thrombus obstructing the microbranches of the portal venous vein [31]. Chou et al. [32] studied 60 HCC specimens with histopathological evidence of MVI, and 40 showed evidence of focal extra-nodular extension. In 36 of 40 HCC specimens with focal extra-nodular extension, non-smooth margins on CT images were located in the same octant as MVI in the histopathological specimens. This finding is consistent with our research and other radiological studies, which reported that the non-smooth tumour margin is an MVI-related risk factor [33, 34].

Some studies have shown that tumour size and an incomplete tumour capsule are also independent risk factors for predicting MVI [35, 36]. These two variables were essential in the clinicoradiological model in this study, but they were not included in the combined model. The high correlation between radiomics features and the two radiological variables may explain this finding. The correlation coefficient heatmap showed that the tumour size and tumour margin are highly correlated with some radiomics features.

The OR of the SZN from the DWI was higher than that of other radiomics features in both the radiomics model and the combined model in the validation set. SZN measures the variability of size zone volumes in the image. Jiang et al. [37] found that the SZN from the PP of enhanced CT is an essential feature for predicting MVI in HCC. Ma et al. [38] reported that the Maximum from the AP and delayed phase of CT was another important MVI-predicting factor. Maximum is the maximum grey level intensity within the ROI. Although the above radiomics features were obtained from contrast-enhanced CT, the SZN and Maximum may be relatively stable for predicting MVI. Most of the radiomics features in the radiomics model and the combined model are matrix-based texture features, and texture features can reflect the heterogeneity of the tumour; therefore, MVI may be related to the heterogeneity of the tumour [31, 39].

Multifocality was not a significant factor affecting MVI in this study. Thus, the cases with multiple lesions were not excluded in this study. We only selected the largest lesion to draw ROI, which may confound the possible effects of multiple center carcinogenesis on outcomes. The prediction model constructed by Yang et al. [21], which combined AFP, non-smooth tumour margin, peritumoural enhancement, HBP T1WI signature, and HBP T1 map signature based on Gd-EOB-DTPA-enhanced MRI, reached an AUC of 0.861 in the validation set. The AUC of the combined model of this study in the validation set was 0.826, which was lower than that of the above study [21]. This might be explained by the fact that our study only extracted features from the largest section of the tumour because of the huge workload. Therefore, some additional information might have been missed. Nevertheless, combining different clinical or radiological variables has a significant effect on the outcomes. In addition, the combined model in our study included variables selected from the combined data, and was thus different from other combined models constructed by merging the variables included in a clinical model or radiomics model. Gitto et al. [40] found that 3D and 2D MRI-based radiomics analyses of cartilaginous bone tumours provide similar rates of stable features, and the 2D approach is easier to implement in clinical practice. Thus, single segmentation of HCC may provide relatively equivalent value for predicting MVI, which may help reduce the workload. Although we used one section to extract radiomics features, the nomogram risk-prediction model based on the combined model still showed good discrimination and value.

This study has some limitations. First, this study used a small sample size, and it was a retrospective, single-centre study without external validation and lacked robustness. Second, this study delineated the ROI at the maximum cross-section of the tumour other than the whole tumour volume, and manual delineation could be affected by the subjectivity of radiologists. Third, we only assessed the intra-observer agreement, and the failure to evaluate inter-observer agreement might affect the stability of the results, given that different observers are subjective in delineating the ROI. Fourth, this study selected essential features from all the sequences but did not compare the value of a single sequence or the combination of different sequences, as the features selected from a single sequence might not always have value in a model based on multiple sequences and the features in a multi-sequence model may not be stable in a single-sequence model [29].

## Conclusions

In summary, two semantic features, peritumoural hypointensity and tumour margin, which are highly correlated with MVI combined with multi-sequences radiomics features have potential value in predicting MVI preoperatively, and the nomogram based on the combined variables can intuitively provide the probability of MVI and guide clinical decisions.

## Supplementary Information

**Additional file 1:** Raw data.

## Data Availability

The data and materials used and/or analysed during the study are available from the corresponding author on reasonable request.

## Abbreviations

Gd-EOB-DTPA: Gadolinium-ethoxybenzyl-diethylenetriamine pentaacetic acid
MRI: Magnetic resonance imaging
MVI: Microvascular invasion
HCC: Hepatocellular carcinoma
CT: Computed tomography
ROC: Receiver operating characteristic
AUC: Area under the ROC curve
HBP: Hepatobiliary phase
HBsAg: Hepatitis B surface antigen
AFP: Alpha-fetoprotein
ALT: Alanine-aminotransferase
AST: Aspartate-aminotransferase
ALB: Albumin
TBIL: Total bilirubin
DBIL: Direct bilirubin
PLT: Platelets
PT: Prothrombin time
INR: International normalised ratio
T2WI: T2-weighted imaging
T1WI: T1-weighted imaging
AP: Arterial phase
PP: Portal venous phase
TP: Transitional phase
ROI: Region of interest
DWI: Diffusion-weighted imaging
ICCs: Intraclass correlation coefficients
OR: Odds ratio
GLSZM: Grey-level size-zone matrix
GLRLM: Grey-level run-length matrix
GLCM: Grey-level co-occurrence matrix
NGTDM: Neighbouring grey-tone difference matrix
GLDM: Grey-level dependence matrix
DV: Dependence Variance
IDN: Inverse Difference Normalized
SALGLE: Small Area Low Gray Level Emphasis
SAHGLE: Small Area High Gray Level Emphasis
SZN: Size Zone Non-uniformity
